# “Who’s on Backup?”—Exploring Internal Medicine Residency Backup Coverage Systems: Findings from a National Survey

**DOI:** 10.1007/s11606-026-10486-4

**Published:** 2026-05-18

**Authors:** Emily S. Wang, Joel C. Boggan, Emily Leasure, Daniel Kim, Lydia Redway, Erica N. Johnson, Patricia F. Kao, Steven J. Durning, Mark Rasnake

**Affiliations:** 1 South Texas Veterans Health Care System, San Antonio, TX USA; 2 UT Health San Antonio Long School of Medicine, San Antonio, TX USA; 3 Duke University School of Medicine, Durham, NC USA; 4 Mayo Clinic Rochester, Rochester, MN USA; 5 University of California, Riverside School of Medicine, Moreno, CA USA; 6 Alliance for Academic Internal Medicine, Alexandria, VA USA; 7 Johns Hopkins School of Medicine, Baltimore, MD USA; 8 American Board of Internal Medicine, Philadelphia, PA USA; 9 Washington University School of Medicine, St. Louis, MO USA; 10 Uniformed Services University of the Health Sciences, Bethesda, MD USA; 11 Naples Comprehensive Health, Naples, FL USA

**Keywords:** graduate medical education, physician workforce, attitude of physicians, illness behavior

## Abstract

**Background:**

Little is known about backup coverage systems for clinical coverage when resident physicians call in for acute absences.

**Objective:**

To define prevalence, core characteristics and explore challenges of backup coverage systems in Internal Medicine (IM) residency programs.

**Design:**

A nationally representative, cross-sectional survey of US IM program directors (PDs) explored “backup” coverage systems.

**Participants:**

PDs of 466 IM residency programs accredited by the Accreditation Council for Graduate Medical Education responded during August–December 2023.

**Main Measures:**

Responses were analyzed using inferential statistics and thematic analysis of an open-ended question.

**Key Results:**

Response rate was 57.1% (266 of 466). IM programs reported formal (92.9%, 247/266) or informal (6.4%, 17/266) backup systems. Most (64.3%, 169/263) felt use of the backup system had increased since the COVID-19 pandemic. Over 90% of programs listed acute illness, family emergencies, fatigue/impairment, and bereavement as reasons for backup coverage. Most programs (> 90%) provided coverage for absences from the inpatient ward, ICU services, and/or night float; however, 40.2% covered absences in primary care clinics. Most programs (87.4%, 229/262) had residents on backup during other clinical/educational rotations. Fewer than 20% of programs compensated residents for backup, namely time off and decreased likelihood of pull for future coverage. Only 15.4% (8/52) of programs provided monetary compensation. For long-term absences, 82.7% (220/266) of programs coordinated trades and 35.7% (99/266) used backup. Six themes characterized challenges of backup coverage: healthcare resources, call-ins culture change, balancing equity, patient care versus education, administrative burden, wellness/ burnout focus, parental/extended leave requirements. IM PDs expressed competing demands with backup systems.

**Conclusions:**

While backup systems are nearly ubiquitous in IM residency programs, PDs report significant strains in these systems including possible cultural shift in illness definition, workload imbalances post-COVID, and prioritization of inpatient coverage over primary care. System-level changes are needed to improve backup coverage systems.

**Supplementary Information:**

The online version contains supplementary material available at 10.1007/s11606-026-10486-4.

## INTRODUCTION

Physicians have faced unprecedented challenges providing care in the years since the onset of the COVID pandemic.^1^ Studies prior to the pandemic showed a prevalence of physician burnout close to 50%, with physicians at the frontline of patient care, such as those in family medicine and general internal medicine at the highest risk.^1^ Later work has shown covering co-workers who were out for sick leave during the COVID pandemic has been an additional challenge on already stressed systems.^2^ In addition, nearly two-thirds of pediatricians met at least one dimension of burnout post-pandemic vs. less than half pre-pandemic.^3^ These and other challenges have the healthcare community recommending creation of workforce reserves to relieve those on duty prior to exhaustion.^2^ One way to address workforce reserves is through coverage systems, which are vital to maintain staffing for attendings and residents alike.^4^ However, the literature on systems for transferring duties when colleagues require coverage is limited.^5^ Residency program coverage systems have been cited as a model for attending physician groups, but evidence describing residency systems is limited to small, single-site studies.^5–7^

Accreditation Council for Graduate Medical Education (ACGME) Common Program Requirements require residencies to have coverage systems that “must be implemented without fear of negative consequences” and cover absences for “…fatigue, illness, family emergencies, and parental leave”.^8^ Such coverage must be provided without an “expectation of reciprocity”.^8^ Thus, residency programs have created ad hoc systems, popularly termed “jeopardy”, “sick pull”, “at risk” or “backup”; hereafter referred to as backup coverage systems, for residents who require coverage for various reasons.^6,7^ The need for and lack of evidence-informed backup systems have several potential consequences. These systems have been suggested as a cause of resident “sickness presenteeism”, whereby individuals continue working despite illness so as to not burden colleagues with additional responsibilities.^9–12^ Inadequate coverage systems are also cited as a source of burnout, and a recent single-site study demonstrated that implementation of a backup system can improve resident satisfaction.^6^

In this study we conducted a national cross-sectional survey of Internal Medicine (IM) residency program directors (PDs) to (a) define the prevalence of backup systems in IM residency programs, (b) describe core characteristics of backup systems, including how programs provide coverage for absent residents; rotations requiring coverage; reasons for coverage needs; and types (if any) of compensation; and (c) understand the challenges IM PDs face in managing these systems. We strive to provide residency leaders’ foundational knowledge on residency backup systems.

## METHODS

### Study Setting and Participants

The Association of Program Directors in Internal Medicine (APDIM) represents over 12,000 academic medicine educators and administrators. The APDIM Survey and Scholarship Committee oversees the development of an annual research survey of IM PDs. In April 2022, a call for thematic topic proposal submissions (for the 2023 Annual Survey) was disseminated online to all roughly 4,600 APDIM physician-members. In July, the 14-member (residency program or associate program directors) APDIM Survey Committee blind-reviewed 21 section topic proposals, scored them on merit and relevance, and invited seven topic authors to submit complete survey section proposals. In October, the committee blind-reviewed the complete proposals and selected “Internal Medicine Residency Back-Up Coverage Systems” as one of three survey topics for inclusion in the 2023 Annual Survey.

The 2023 Annual Survey was disseminated to PDs from all 466 member residency programs. At the time of the study, APDIM member programs represented 80.0% (466/586) of residency programs with ACGME accreditation.

### Survey Instrument

In December 2022, APDIM Survey Committee members were appointed as section-development co-contributors to work with non-committee section authors to create the research team for the backup coverage topic section. From March through early May 2023, the original questions were revised through an iterative process, the committee pretested the complete instrument for construct validity and clarity, and further content revisions were made. In mid-May, the project personnel (LR) programmed the instrument in the Qualtrics Surveys platform.^13^ From mid-June through mid-July 2023, the web survey was pilot tested for content validity evidence by the committee and 11 APDIM members consisting of experts in GME (blinded to the committee). AAIM Surveys staff (LR), trained in best practices for survey methodology, also reviewed and commented on the complete survey. Section leads were required to address all relevant pilot test comments and feedback, and final revisions were made to the instrument. The study (#2023-0236-DFT) was deemed exempt by Pearl IRB (U.S. DHHS OHRP #IRB00007772) under 45CFR46.104(b)(2): (2).

The survey included 78 questions (some with subparts) with 17 questions on backup systems (Appendix 1). The survey launched on August 10, 2023, and closed on December 12, 2023. Participants received a unique, individualized link to the survey. Six email reminder messages were sent to nonrespondents. The email invitation and all email reminders included opt-out links for individuals who did not wish to participate in the survey. No incentive was provided for participation.

### Statistical Analysis

Data analysis was conducted in *Stata SE 18.0* by LR. Before de-identifying the final responses,^14^ the study dataset was appended with residency program data (using the ACGME program identification number) from sources including the U.S. Census Bureau (to identify geographic regions).^15^ Characteristics such as number of approved resident positions were obtained from the ACGME *Accreditation Database System (Public)*.^16^ Statistical significance was designated with an alpha level set to p ≤ 0.05. We tested for possible associations between categorical variables using the Adjusted Wald (Pearson) Chi-Square test of association, a test statistic that is more sensitive to survey data stratified by key population characteristics (e.g., residency program type). Due to the non-normal, nonparametric distribution of continuous variables, we used the two-sample Wilcoxon rank-sum (Mann–Whitney) test. Additional statistical analysis strategies are discussed in Appendix (Appendix 2).

### Thematic Analysis

A thematic analysis using Braun and Clarke’s approach was conducted on the open-ended survey question, “What has been the most challenging aspect(s) of back-up coverage for your program?”.^17^ EW, JCB, MR, and SJD independently reviewed all responses. All the responses were analyzed at the utterance level (fewest words that capture the idea) and codes were generated using an inductive approach. Codes were grouped and categorized into initial themes. Themes were discussed and refined through a series of meetings, reaching complete consensus on themes and sub-themes. We reached thematic saturation, and representative responses were chosen for each theme. Using a post-positivist paradigm, we also quantified themes at the utterance level to assess possible trends in quantification of challenges.

## RESULTS

The survey response rate was 57.1% (266/466). While our analysis determined that the survey-eligible population was representative (Table 1), there was slight respondent over-representation of university-based programs (37.2%, 99/266 vs. 29.4%, 137/466 for the population; p = 0.049) and programs that require rotations at one or more Veterans Affairs (VA) hospitals (39.5%, 105/266 vs. 33.1%, 154/466 for the population; p = 0.011). The median accreditation year for responding programs was earlier than that of all survey-eligible programs: 1968 (IQR: 53) vs. 1973 (IQR: 58), respectively (p = 0.004). Finally, programs with a larger number of full-time physician faculty were over-represented compared to the complete survey population (median: 39 [IQR: 141] and median: 24 [IQR: 104], respectively; p = 0.038). Due to survey-conditional logic or item non-response, denominators for certain questions will not sum to the total respondent number (Table 1).

**Table 1:** Essential Characteristics of Responding and Nonresponding Internal Medicine Residency Programs: 2023 APDIM Survey of U.S. Internal Medicine Residency Program Directors

	Respondents (*n *= 266)	Nonrespondents (*n* = 200)	Total (*n* = 466)	
	No. (Column %)	No. (Column %)	No. (Column %)	*P*-value^a^
Program Type (AMA-FREIDA)				
* University-based*	99 (37.2)	38 (19.0)	137 (29.4)	**0.049**
* Community-based*	36 (13.5)	53 (26.5)	89 (19.1)	0.071
* Community-based, university-affiliated*	128 (48.1)	106 (53.0)	234 (50.2)	0.611
* Military-based*	3 (1.1)	3 (1.5)	6 (1.3)	0.793
Census Region (U.S. Census Bureau)^b^				
* Midwest*	47 (17.7)	54 (27.0)	101 (21.7)	0.222
* Northeast*	81 (30.5)	52 (26.0)	133 (28.5)	0.599
* South*	99 (37.2)	59 (29.5)	158 (33.9)	0.441
* West*	39 (14.7)	35 (17.5)	74 (15.9)	0.412
Accreditation Status (ACGME)				
* Continued or continued with warning*	256 (96.2)	189 (94.5)	445 (95.5)	0.475
* Initial or initial with warning*	10 (3.8)	11 (5.5)	21 (4.5)	
VA hospital rotations required: Yes (ACGME)	105 (39.5)	49 (24.5)	154 (33.1)	**0.011**
Offers preliminary positions: Yes (AMA-FREIDA)	187 (70.3)	123 (61.5)	310 (66.5)	0.138
Offers J-1 visa sponsorship through ECFMG: Yes (AMA-FREIDA)	215 (81.4)	143 (72.2)	358 (77.5)	0.069
	**Mean (SD),** **Median (IQR)**	**Mean (SD), Median (IQR)**	**Mean (SD), Median (IQR)**	***P*-value**^**c**^
Program size: No. ACGME approved positions (AY 2022–2023)	69.4 (43.2), 53 (63)	58.2 (35.8), 45 (35.5)	64.6 (40.5), 50 (46)	0.256
ABIM rolling pass rate 2020–2022 (%); n = 244, n = 165, n = 409	89 (10.7), 91.5 (10)	87.4 (11), 90 (15)	88.3 (10.9), 91 (11)	0.250
Program director tenure (years) as of 2023 (ACGME)	5.5 (5.4), 4 (6)	5.5 (5.8), 4 (6)	5.5 (5.5), 4 (6)	0.854
Program original accreditation year (ACGME)	1977.8 (25.3), 1968 (53)	1986.9 (27.2), 1980 (57)	1981.7 (26.5), 1973 (58)	**0.004**
Physician faculty Full-time (FREIDA)	103.9 (151.1), 34 (141)	64.2 (119.2), 18 (53)	86.8 (139.6), 24 (104)	**0.038**

*APDIM* Association of Program Directors in Internal Medicine, *AMA-FREIDA* American Medical Association Residency and Fellowship Database, *ACGME* Accreditation Council for Graduate Medical Education, *ABIM* American Board of Internal Medicine, *ECFMG* Educational Commission for Foreign Medical Graduates, *VA* Veterans Affairs, IQR=interquartile range, *SD* Standard Deviation

Table includes the variables that explained the most survey population variance: quantile (median) regression model (dependent variable: “total number of filled ACGME-approved resident positions”); pseudo R2 = 0.41; raw sum of deviations = 6,833.5

^a^Adjusted Wald (Pearson) test of association (one degree of freedom) used to compare categorical variables

^b^Collapses two programs from U.S. territories into “West,” due to small cell sizes/data confidentiality

^c^K-sample equality-of-medians test (continuity corrected Pearson Chi-Square) [mean and SD reported for illustration]

**Figure 1:**
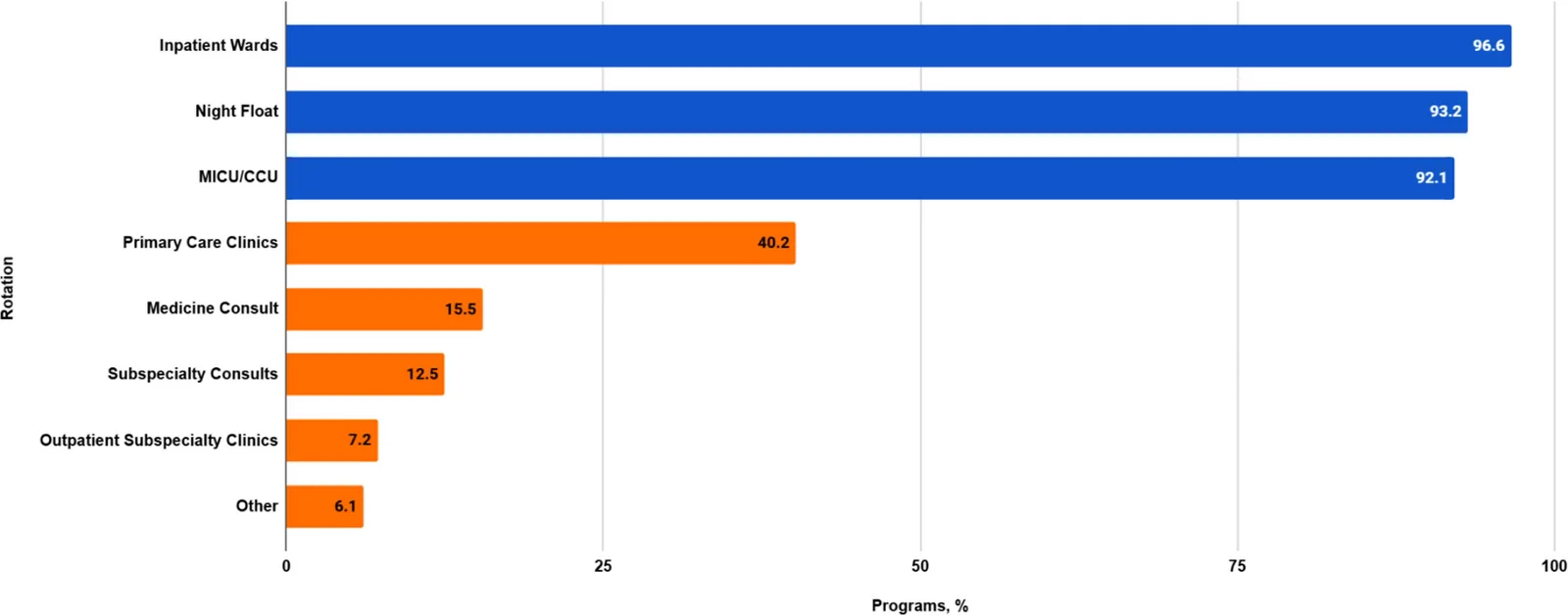
“Which IM rotation(s) require coverage to be secured when a resident has an unexpected absence? *Check all that apply*.” Multiple responses allowed and total will exceed 100%. Two hundred sixty-four respondents, total responses n = 959. Inpatient ward (96.6%, 255/264), intensive care unit (ICU) (92.1%, 243/264), night-float rotations (93.2%, 246/264), primary care clinics (40.2%, 106/264), medicine consults (15.5%, 41/264), sub-specialty consult rotations (12.5%, 33/254), outpatient sub-specialty (7.2%, 19/264), other (6.1%, 16/264).

Most programs (> 99%) have a formal (92.9%, 247/266) or informal (6.4%, 17/266) backup system to cover resident clinical absences. Formal systems have a set of structured guidelines (e.g. who is to provide coverage, which rotations are covered) versus informal systems where there is no set of structured guidelines and/or policies. Two programs have no backup system (0.8%). Larger programs (mean = 57.3 categorical residents; SD = 42.2) were significantly more likely to have formal backup systems than smaller programs (mean = 35.9; SD = 32.9; p = 0.0006). Most (64.3%, 169/263) PDs felt that use of backup systems had increased greatly (33.5%, 88/263) or somewhat increased (30.8%, 81/263) since the onset of the COVID-19 pandemic. Very few (6.8%) felt it had decreased. More than 90% of programs with backup systems provide coverage when residents are absent from the inpatient ward (96.6%, 255/264), the intensive care unit (ICU) (92.1%, 243/264), and/or night float rotations (93.2%, 246/264); a smaller percentage cover absences from the primary care clinic (40.2%, 106/264), while few cover consulting or non-primary care ambulatory experiences (Fig. 1). Roughly 40.5% (106/262) of IM programs require their IM residents to cover non-IM residents assigned to medicine services at least some of the time.

While most programs with a backup system (87.4%, 229/262) assign residents to the coverage pool who have other clinical/educational responsibilities, a fraction (31.3%, 82/262) has at least some residents in the coverage pool without other assigned responsibilities. Over 95% (218/229) of programs with assigned residents with other scheduled clinical/educational responsibilities utilized residents from elective rotations, but other experiences, in descending order of frequency, include research rotations (62.0%, 142/229), required sub-specialty rotations (41.9%, 96/229), and educational time (e.g. board review 32.3%, 74/229). Of note, residents assigned to primary-care clinic were part of the coverage pool at 24.9% (57/229) of those programs.

**Figure 2:**
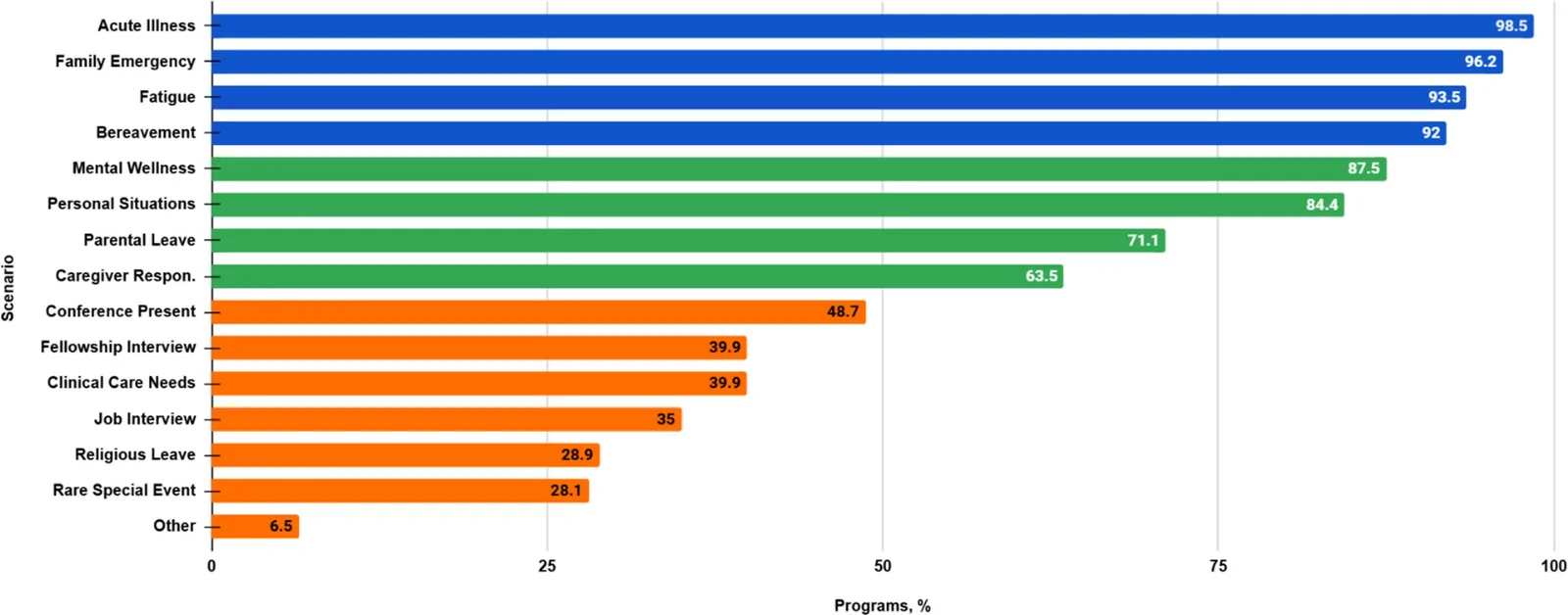
“For what scenario(s) would your program activate the coverage system? Check all that apply.” Multiple responses allowed and total responses will exceed 100%. One non-respondent, 263 of 264 respondents, total responses n = 2403. Acute illness (98.5%, 259/263), family emergencies (96.2%, 253/263), fatigue/impairment (93.5%, 246/263), bereavement (92.0%, 242/263), mental well-being/burnout (87.5%, 230/263), non-health-related personal emergencies (84.4%, 222/263), parental leave (71.1%, 187/263), child/caregiver responsibilities (63.5%, 167/263), conference attendance (48.7%, 128/263), clinical/patient care needs (39.9%, 105/263), fellowship (39.9%, 105/263), job interviews (35.0%, 92/263), religious services/holidays (28.9%, 76/263), rare special events (28.1%, 74/263), other (6.5%, 17/263)

Over 80% of programs list acute illness, family emergencies, fatigue/impairment, bereavement, mental wellness and/or personal situations (e.g. flooding) as reasons to utilize their backup system (Fig. 2). Other circumstances covered in descending order include mental well-being/burnout, non-health-related personal emergencies, parental leave, and child/caregiver responsibilities, conference attendance, religious services/holidays, clinical/patient care needs, and fellowship or job interviews (Fig. 2). If longer-term absences were expected, such as parental leave, most programs (82.7%, 220/266) prioritized program-arranged or resident-arranged schedule swaps, while 35.7% (99/266) used the existing backup system.

While most programs tracked metrics of their backup system, this was not universal, and metrics tracked varied by program. The resident requiring coverage (84.0%, 221/263) and the resident providing coverage (79.9%, 210/263), total number of days of coverage required (73.0%, 192/263) or provided (68.8%) were the most frequently cited. Fewer than half tracked which rotations required coverage or which rotations were impacted by having residents pulled. Finally, 38.8% (102/263) of programs tracked primary care clinic cancellations that resulted from coverage pulls.

Overall, 57.2% (151/264) of programs with a backup system required payback in some circumstances, and 8.0% (21/264) in all circumstances. Of the programs requiring payback, 62.3% (94/151) of PDs felt requiring payback reduced the likelihood of residents requesting coverage at least to some extent. Fewer than 20% (19.7%, 52/264) of programs compensated residents who were pulled for coverage, with the most common forms of compensation being compensatory time off and decreased likelihood of future pulls. Monetary compensation was uncommon, cited by 15.4% (8/52) of programs. If a backup system is exhausted, many programs (51.3%, 135/263) requested volunteers and/or asked attendings to cover (41.8%, 110/263).

Our thematic analysis of open-ended responses on challenging aspect(s) of backup systems analyzed > 6400 words from 223 of 264 respondents who have backup systems (41 did not answer). The following themes were identified from the most frequent to the least frequent based on quantitative statement count—healthcare resources, changes in call-in culture, balancing equity, patient care versus education, administrative burden, wellness and burnout focus, and parental and extended-leave requirements with definitions and representative statements (Table 2). We did not identify any new themes in the final 25% of the open-ended responses from the respondents, thus thematic saturation was reached.

**Table 2:** Thematic Analysis of comments to “What has been the Most Challenging Aspect(s) of Backup Coverage for your Program?”

Theme	Definition	Examples/Quotes
Healthcare Resources (24.2%, 86/355) Materials, personnel, facilities and funds to provide healthcare with a focus on manpower and funding Sub-theme: Non-medicine services coverage	Manpower challenges relating to the size of the program and mismatch with the medicine service lines/teams and expectations by the department/sub-specialty services needing coverage by the residency program Sub-theme Non-medicine services coverage: Challenges with the non-medicine residents rotating on medicine services due to ACGME requirements (to include preliminary years, transitional year, emergency medicine, family medicine, psychiatry, etc.) and medicine program need to provide backup coverage for non-medicine residents on service	“ small program with limited backup options; in addition historic institutional precedent for all subspecialty consult teams to have resident coverage year round” “ IM residents (historically) felt a deep sense of unfairness when off-service residents (TY, EM, FM) took vacations from medicine and ICU teams without providing internal coverage. The IM program would have to find moonlighters or use Jeopardy for these planned absences. During COVID, IM Jeopardy was also used if off-service residents were home awaiting COVID test results or confirmed COVID +. The burden of coverage felt very unfair.”
Changes in Call-ins Culture (20.6%, 73/355) Sub-theme: COVID-19 impact re-defines “sick” and coverage needs	Changes in “calling in sick” related to the frequency and/or timing. In addition to changes in reasons to “calling in sick” to include COVID/other illness, flight delays, increased use of days around “golden weekends” and holidays, sick leave seen as “paid time off” Sub-theme COVID-19 impact re-defines “sick” and coverage needs: shifted the definition of illness due to quarantine days differences within different systems, mild symptoms calling in sick	“ I have been APD or PD for 20 years. The back up system is used much more frequently (literally 10x) more frequently over the years.” “ The number of residents using their sick leave has increased exponentially. We try to balance a culture of wellness with promoting good work ethic, while refraining from asking evidence for all sick days e.g. how does one quantify migraine severity etc. This has been one of my toughest challenges as a PD in the post-covid era.” “ Before Covid, almost no one called in sick, which was not necessarily a good thing. After covid, people call in for almost any symptom, even just sniffles. We have tried to restructure our backup system so that we are more judicious about what constitutes a reason to be out while not going back to the precovid “never call in sick” mentality.” “More absences. More absences on weekends or days flanking weekends. More absences on rotations that are clinically heavier.”
Balancing Equity (18.0%, 64/355)	The balance of fairness and justice as it pertains to those using sick leave and those pulled to cover the sick leave. There are stressors regarding who provides judgement on appropriateness of call-ins, accountability, and use by “high frequency users”. When residents are called-in to cover, the parity of “payback systems” and residents pulled from electives with the emphasis on service over learning	“ We cannot place “value” on some requests and not others, so more nebulous requests for “emergencies” or “burnout” we cannot question and my fear is that some residents are more inclined to pull for these reasons than others and it feels somewhat inequitable, but we accommodate most requests as an honor system of them being requested appropriately.” “Perceived Equity. Residents who have a high threshold to call jeopardy expressing resentment towards residents who have a low threshold and call jeopardy more frequently. At the same time Low threshold residents feeling that their needs are valid and needed.” “managing feelings of inequity among residents despite enormous effort to ensure things are as equitable as possible”
Patient Care vs. Education (12.4%, 44/355) Sub-theme: Primary Care/outpatient deprioritization Sub-theme: Fellowship competitiveness	There is the balance of service/patient care and education and professional development of the residents are concerns of mismatch inpatient care over primary care/outpatient clinics. Coverage needs such as nights, weekends, holidays needing in-house coverage by the program are required. This creates transitions of care (TOC) challenges with interruptions in team dynamics, continuity of patient care Sub-theme Primary care deprioritization: Residents on primary care/outpatient rotations are pulled to cover inpatient rotations causing cancellation of clinic appointments Sub-theme Fellowship competitiveness: Residents are pulled from elective experiences which impacts fellowship and the increased competitiveness of fellowship with interviews and conference attendances/presentations	“ Residents are being pulled from continuity clinic and their electives to cover colleagues which is demoralizing especially if the elective is important for future career. In addition, we estimate that over 500 patient care appointments were cancelled due to pulling residents in for back up.” “Pulling folks from continuity clinic to cover inpatient such that outpatient patient care can be affected.” “… increased number of fellowship interviews; increased scholarly activities and number of posters accepted at national meetings”
Administrative Burden (10.4%, 37/355)	The challenges to manage complex schedules to include; but not limited to time, stress, skilled personnel. Frequently the administrative responsibility is managed by a chief resident to track, monitor, maintain and account for call-ins and paybacks in a complex residency schedule of X + Y, seasonal needs such as illnesses from Dec-Feb and May–June end of transitions. Due to the nature of the work and complexity of the schedule, a chief resident or other program leadership team member is necessary	“ May–June is the hardest time of the year as far as back up utilization is concerned. PGY3s sick leave request skyrocket, domino effect on morale of junior residents. Cycle repeats year after year.” “Residents do not always activate the system properly by directly communicating the need for coverage to program leadership and receiving a response that the message has been received (e.g., direct telephone call or text with a confirmatory response).” “finding appropriate coverage or rotation switch”
Wellness and Burnout Focus(10.1%, 36/355)	The emphasis on wellness of the individual and the choices made in favor of wellness of the individual including prioritization of mental wellness days and increasing importance on wellness by ACGME	“ Different cultures between classes for when to call out. Some classes want to be able to call out for things like mental health days and are ok covering each other for that, but other classes don’t want to call out at all. This has bred animosity between classes in some cases.” “How to handle the requests ‘wellness days’…we’re happy to give them and understand but then residents go for a hike to feel better/be well, post about it on social media, and it becomes an issue.”
Parental Leave/Extended Leave Requirements (4.2%, 15/355)	Implementation of mandatory paid leave related to parental/caregiver 6 weeks (7/1/2022) and other extended medical leave with increased number of residents taking leave and the length of time of coverage taken	“ Increased availability of parental leave (more liberal policy) has stressed program back-up coverage system significantly.” “Residents with children are absent more frequently than those without and it causes interpersonal issues that can be difficult to manage.”

Thematic analysis of the open-ended responses to “What has been the most challenging aspect(s) of backup coverage for your program?”. Over 6400 words from 223 of 264 respondents who respondents who have a backup system (41 did not respond to this question). Total number of utterances identified *n* = 355

*Healthcare resources* were defined as materials, personnel, facilities and funding. This was the most frequently discussed theme. Notable mentions include a mismatch between the size of the program/number of residents available to cover the number of service lines expected by clinical departments, including sub-specialty services. Multiple service lines, particularly ICUs, require 24-h coverage. A sub-theme is the need for non-medicine residents requiring coverage by IM residents. PDs mentioned the challenges around ACGME requirements for non-medicine specialties to have experiences on medicine services; however, when non-medicine residents need leave or take vacation, IM residents would need to cover.

“Multiple residents calling out at the same time. We do not have enough flexibility in the schedule to create layered backup while fulfilling ACGME requirements.”

*Change in call-ins culture* referred to the frequency, timing, and the reason for residents calling in sick and was the next most identified theme. Responses highlight that the perceived frequency of call-ins has increased multifold. Call-ins also occur around days off (e.g. before or after vacation or weekend), and the shift in culture of “sick days” to paid time off. Reasons for calling in also include non-illness reasons (e.g. flight delays). A sub-theme identified was that COVID-19 impact redefined “sick” and coverage needs and how this has changed illness definitions, with symptoms previously considered mild now used for call-ins and the number of days needed for backup coverage has increased.

“Covid-trained residents to call out sick for every minor complaint, now need to reinforce it is an EMERGENCY system.”

*Balancing equity* was defined as the fairness and justice of residents who call in sick and the residents who provide coverage. This includes challenges of adjudicating the appropriateness of call-ins, maintaining accountability in “payback” systems, and monitoring the use of call-ins by residents perceived as “high utilizers”.

“The indications for calling for backup is difficult to determine without infringing on the privacy of the resident. But it is also difficult to assess the standards of professionalism required of physicians without doing so.”

*Patient care versus education* theme was defined as the difficulty balancing the residents’ needs and prioritization of caring for patients, especially during holidays, nights and weekends that are perceived to be less focused on learning and more on service needs of inpatient settings. This is further emphasized in the sub-theme of inpatient services requiring coverage by residents being pulled from primary care and outpatient experiences—thus, ambulatory care is seen as less important for education. An additional sub-theme is fellowships: a) competitiveness of fellowships has placed stress on residents being removed from elective experiences (e.g. less time with key mentors); b) requesting more time off for fellowship interviews, sub-specialty conference attendance, and presentation of scholarly activity.

“Pulling residents from electives which are an important aspect of their education.”

The *administrative burden* required to coordinate a backup system, requiring personnel to understand, maintain, organize and track the needs of service lines, was identified. Management of this system is generally a chief resident or a program leadership member due to schedule complexities, including X + Y blocks, seasonal coverage needs such as respiratory viral season, and yearly-recurring transitions of residents to fellowships.

“Maintaining accurate records of absence and coverages.”

With the recent emphasis on *wellness and burnout focus* in medical education*,* PDs stated individuals may have different approaches to mental wellness, as some residents may request coverage for mental wellness days versus others who do not but are then asked to cover.

“Getting the residents to understand that there are some days when you come to work even when unexpected events are happening at home or when you don’t feel your best, and that doing this ultimately gives you greater satisfaction with your work and less burnout in the long run.”

With the institution of ACGME mandatory paid *parental leave*, this and extended sick leave requirements are cited as challenges for program directors to arrange coverage due to the increased number of residents taking leave and the amount of time taken.

“Accommodating need for more sick leave, expanded parental leave, and expanded medical leave with a fixed FTE.”

## DISCUSSION

Our nationwide study of IM residency programs finds that nearly all programs have backup systems. Our quantitative findings were consistent across institutions, including: (a) reasons for using backup systems; (b) coordination of swaps to avoid using backup systems for long-term absences; (c) prioritization of coverage for inpatient rotations over primary care experiences; (d) backup coverage assigned to those with other scheduled rotations; (e) and infrequent provision of compensation for those pulled to cover. However, the qualitative results demonstrated that most IM PDs found their backup systems challenging. IM PDs are concerned with the balance of resources, the lack of reserve personnel, and mismatched requests and requirements for coverage by departments and hospitals. Other themes included a perceived shift in the culture and increased frequency of call-ins, a need for equity in backup systems, and prioritization of wellness.

Our study also highlights multiple areas in need of further discussion. First, it is laudable that IM residency programs have created systems that prioritize patient care responsibilities without clear guidelines on managing these systems. Sharing data about backup systems across institutions can highlight best practices. While our residents are a versatile workforce, our data show these systems are frequently running on “thin margins” and are not a limitless supply of human resources.

Second, our data hint at a cultural shift in medicine. Prior literature suggests “sick presenteeism” occurs in resident and attending physicians as a cultural norm.^5,9–12^ In this study, most IM PDs report that use of backup systems increased after the COVID-19 pandemic, with a concomitant change in the definition of illness. While a shift from a previously unhealthy culture of “working ill” is beneficial, existing backup systems have not yet accommodated this change. Similarly, the ACGME recently mandated six weeks of paid parental leave. While this is a laudable change, the timing of its deployment post-COVID pandemic may have exacerbated challenges related to already taxed backup systems.^18^

Third, our data highlight longstanding tensions between service versus education in graduate medical education.^19–21^ Specifically, IM PDs struggled to protect educational activities while maintaining clinical service coverage. Residents on elective rotations form the backup coverage pool over 95% of the time. These electives are often critical experiences for those seeking entry into highly competitive fellowships.^22^ In addition, our analysis found inpatient rotation prioritization over primary care continuity experiences. Not only did fewer than half of IM PDs require coverage when a resident is absent from primary care clinic, but residents on primary care rotations were included in the coverage pool by one quarter of respondents. There may be many reasons inpatient rotations are prioritized over primary care clinics, such as the ability to reschedule outpatients, the acuity of illness of inpatients, and some inpatient ward teams entirely staffed by residents; however, such practice risks sending the message that outpatient care is not valued as highly as inpatient care.

Fourth, we found navigating wellness to be a consistent concern. The Institute of Medicine has cited physician fatigue as a patient safety concern, leading to changes in duty hours in 2003 and 2011.^23^ When we consider these changes and the current emphasis on wellness, this may explain why some IM PDs perceive changes in the culture of sick leave use. Some suggest residents may view these days as additional “paid time off” to be used or lost. Addressing the stated wellness needs of trainees while managing the complexity of schedules, tracking leave requests, and other administrative tasks may be contributing to IM PD burnout.^24^ Balancing the wellness of individual residents, the work culture of the program, and the needs of the patients is a source of moral distress for PDs. Some have described the challenge of maintaining the professionalism ideals of the medical field in a time of concern for “erosion of professionalism or a new norm” in medicine.^25^

This study is not without limitations. Survey research inherently is subject to some degree of bias including non-response bias, respondent error, and recall bias. The 57.1% survey response rate was broadly representative of the underlying population of IM residency programs eligible to complete the survey, albeit with slight over-representation of “older”, university-based programs. Not all respondents answered all the questions. There are also many areas that could be further explored to include topics such as correlations with program demographics and backup coverage systems, how residency programs determine when “payback” is required, and are more residents are using most or all their sick leave. We also believe, in light of recent resident unionization efforts, that while backup coverage systems may be alluded to, this should be an area for formal discussions and policies.^26,27^ In addition, our qualitative analysis was limited by solely including written responses, although analyzing written responses using thematic analysis is considered an appropriate approach.^16^ We also chose to use a post-positivist paradigm to quantify the responses to give the reader a sense of frequency. A future study could approach this research question using interviews or focus groups.

## CONCLUSIONS

Our study is the first to identify the prevalence, key characteristics and challenges of backup systems in IM residency programs. While backup systems are nearly ubiquitous, IM PDs expressed a number of management challenges. We believe there is an opportunity to consider possible interventions and recommendations at multiple levels using the micro-meso-macro healthcare systems framework. At the micro/individual level, while individuals have differing perspectives of wellness, we believe there should be clear expectations for personal and professional physician responsibility surrounding use of backup systems for bona fide needs such as acute illness. At the meso-level, these data should be shared as baseline information for additional investigation, as well as best practices and guidelines. We also believe programs should consider these findings as an opportunity to emphasize the importance of primary care experiences to train the primary care workforce and provide comprehensive patient care.^28^ Finally, we believe the emphasis should be placed on the macro-level recommendations to include stakeholders in graduate medical education, clinical, and healthcare system leadership to create policies and develop best practices for backup systems.

## Supplementary Information

Below is the link to the electronic supplementary material.

Supplementary file1 (DOCX 130 KB)

## Data Availability

The data that informed this research survey study include a limited number of U.S. internal medicine residency program characteristics that were obtained through a data sharing license granted to the study personnel or through databases that are publicly available for querying but are not the property of the study personnel. As per the license or usage terms designated by the parties that maintain ownership of those data, the study personnel may not transfer or publish datasets that contain that information (identified or de-identified), which is intended for descriptive or comparative analyses of the survey data collected by the study personnel but may not be transferred beyond those personnel.

## Declarations

**Ethical Approval**

The study (#2023-0236-DFT) was deemed exempt by Pearl IRB (U.S. DHHS OHRP #IRB00007772) under 45CFR46.104(b)(2): (2).
