# Supplementary material for: “Who’s on Backup?”—Exploring Internal Medicine Residency Backup Coverage Systems: Findings from a National Survey
Source: J Gen Intern Med. 2026 May 18;41(11):3203–12. doi: 10.1007/s11606-026-10486-4 (PMC13493720; doi:10.1007/s11606-026-10486-4)
Supplement: Supplementary file 1 [file 11606_2026_10486_MOESM1_ESM.docx]

**Appendices**

**Appendix 1:**

2023 APDIM Annual Survey of Internal Medicine Residency Program Directors Survey Landing Page and Instrument for Section II: “Internal Medicine Residency Back-Up Coverage Systems”

**Appendix 2:**

Statistical Analysis Strategy

**Appendix 1**

**2023 APDIM Annual Survey of Internal Medicine Residency Program Directors Survey Landing Page and Instrument for Section II: “Internal Medicine Residency Back-Up Coverage Systems”**

**2023 APDIM Annual Survey of Internal Medicine Residency Program Directors**

APDIM collects nationally representative data to provide residency program directors (PDs) with critical information on internal medicine (IM) graduate medical education. **Immediately after submitting your survey responses, you will receive them by email.**

This study (#2023-0236-DFT) is exempt by Pearl IRB (U.S. DHHS OHRP #IRB00007772) under FDA 45 CFR 46.104(d)(2). You are invited to participate as a residency PD whose program was ACGME- accredited *prior to the past Academic Year* (before July 1, 2022) and holds APDIM membership.

**Participation is voluntary:** refusal to participate will not affect your / your program's APDIM membership. The survey software will alert you of any unanswered questions but you may skip those that you cannot / do not wish to answer.

Your responses are confidential. No results containing identifiers of you / your program will be published or disclosed. Alliance for Academic Internal Medicine (AAIM) Surveys staff serve as project leads, manage data collection, and must remove all identifiers upon survey closure.

**If you encounter technical problems or have questions about this survey,** please contact [surveys@im.org](mailto:surveys@im.org) or 703-341-4540. If you feel that your participant rights have not been upheld, contact Pearl IRB at [**info@pearlirb.com**](mailto:info@pearlirb.com) or 317-602-5917.

Expect to allocate about **10 minutes to Section I** and **20 minutes to the remaining sections (possibly less time, depending on your responses)**. **You may exit and return without losing your data.** The unique survey link in your email invitation will return you to where you left off. **DO NOT USE** your browser's **"Back" or "Forward" buttons to navigate: you must use the <BACK and NEXT> buttons at the bottom of each page.** This survey is compatible with most tablet devices, but **smartphone use is discouraged**.

***SURVEY COMPLETION***

1. Unless otherwise specified, this survey applies to the most recently completed academic year (AY). Consider residents enrolled in your training program on **December 31, 2022** as part of that AY.
2. Questions apply to IM residents in a three-year training program and **unless otherwise specified (e.g., Section III) do not apply to** 1. trainees in a transitional year or preliminary program or 2. subspecialty fellows or trainees from programs not included in your ACGME program number.
3. Access to your program characteristics will ease survey completion. **A PROGRAM COORDINATOR OR ASSOCIATE PROGRAM DIRECTOR MAY COMPLETE MOST OF**

**SECTION I.** Share your unique survey URL with caution and please check the information entered by others.

***By clicking below, you acknowledge that your participation is voluntary.***

▢ *Click “PROCEED” (below) to begin*

*Display This Question If “By clicking below…” is EMPTY*

***Do you acknowledge that your participation is voluntary?***

- ***Yes*: *BEGIN survey***
- ***No (you will EXIT and not be able to return)***

*Skip To End of Survey If “NO…” Is Selected*

**Start of Block: Section II. Residency “Back-up, Sick Pull, Jeopardy” Coverage Systems Section II. Internal Medicine Residency Back-Up Coverage Systems**

***Note:*** *“Back-up,” “Sick Pull, “Jeopardy,” and related terms in this section refer to a system that provides coverage when a resident is unexpectedly unable to work.*

**Q1 Does your program have a system to provide resident coverage when a resident is unable to work due to an unplanned / unexpected reason?**

- No: we do not have a system in place
- Yes: a *formal* system with a set of structured guidelines (e.g., who is eligible to provide coverage, which rotations are covered)
- Yes: an *informal* system with steps we apply to provide coverage, but no structured guidelines or policy

*Skip To: Q12 If Q1 = No: we do not have a system in place*

**Q2 Which IM rotation(s) require coverage to be secured when a resident has an unexpected absence? *Check all that apply.***

▢ Inpatient wards

▢ Intensive care units (medical intensive care, cardiac intensive care)

▢ Some or all primary care clinics (resident template or attending template)

▢ Medicine consult rotations

▢ Some or all subspecialty consult rotations (not medicine consults)

▢ Some or all ambulatory subspecialty clinics

▢ Night float / night rotations

▢ Other (please specify):

▢ ⊗*No rotations require coverage*

**Q3 When residents are asked to provide coverage, what rotation(s) / dedicated time might they be “pulled from?” *Check all that apply.***

▢ Back-up designation *without* other scheduled clinical or educational responsibilities

▢ Back-up designation *with* other scheduled clinical or educational responsibilities

▢ Vacation or scheduled leave

*Display This Question: If Q3 = Back-up designation with other scheduled clinical or educational responsibilities*

**Q4 What clinical or educational responsibilities might residents be on when they serve as "back- up?” *Check all that apply.***

▢ Primary care continuity clinic

▢ Required subspecialty rotations

▢ Elective rotations

▢ Educational time (e.g., board exam study)

▢ Research time

▢ Other (please specify):

**Q5 *Excluding* resident-initiated trades / swaps, for what scenario(s) would your program activate the coverage system? *Check all that apply.***

▢ Clinical / patient care needs (e.g., over patient census / caps)

▢ Acute illness (including physical or mental illness)

▢ Fatigue (acute or emergent with concerns for impairment)

▢ Mental wellbeing / burnout

▢ Religious leave / holiday(s)

▢ Family emergency

▢ Parental leave

▢ Childcare or caregiver obligations

▢ Fellowship interviews

▢ Job interviews

▢ A conference presentation requiring travel (regionally or nationally)

▢ Rare special events (e.g., weddings, anniversaries, graduations)

▢ Bereavement (e.g., funerals)

▢ Other personal situations (e.g., house flooding, vehicle problems)

▢ Other (please specify):

**Q6 What does your residency program track (or monitor) for your coverage system? *Check all that apply.***

▢ Resident requiring coverage

▢ Resident providing coverage

▢ Rotation requiring coverage

▢ Rotation resident is pulled from for coverage

▢ Number of days each resident has required coverage

▢ Number of days each resident provided coverage

▢ Continuity clinic cancellations

▢ Other (please explain):

▢ ⊗*Our program does not track (or monitor) the coverage system*

**Q7 Does your program require payback (e.g., more days on future back-up, higher likelihood of future back-up pulls) for residents requesting coverage?**

- No
- Yes: in all circumstances
- Depends on the circumstance as to why coverage was requested

*Display This Question: If Q7 = Yes: in all circumstances*

*Or Q7 = Depends on the circumstance as to why coverage was requested*

**Q8 How do you believe that requiring payback affects a resident's likelihood of requesting coverage?**

- Greatly increases the likelihood
- Somewhat increases the likelihood
- Does not affect the likelihood
- Somewhat decreases the likelihood
- Greatly decreases the likelihood
- *Unsure*

**Q9 Does your program compensate / reward the resident who provides coverage?**

- No
- Yes

*Display This Question: If Q9 = Yes*

**Q10 What is the compensation / reward? *Check all that apply.***

▢ Monetary

▢ Non-monetary (e.g., sports tickets, etc.)

▢ Reduced likelihood of future back-up pulls

▢ Compensatory time off

▢ Other (please specify):

**Q11 If the resident coverage pool is empty / exhausted, how are additional coverage needs managed? *Check all that apply.***

▢ Resident is paid for additional shifts / coverage

▢ Resident volunteers are requested

▢ Resident is designated by the program

▢ Attending(s) / faculty substitute for resident(s)

▢ Chief resident(s) substitute for resident(s)

▢ Advanced practice providers substitute for resident(s)

▢ Resident is pulled from continuity clinic

▢ Other (please specify):

▢ ⊗*No additional coverage is secured (e.g., workload is divided among team members present)*

*Display This Question: If Q1 = No: we do not have a system in place*

**Q12 What does your program do when a resident calls in and is unable to work due to an unplanned / unexpected reason?**

**Q13 What does your program do to cover predicted / planned *long-term absences* (e.g., parental leave, caregiver leave, extended illness)? *Check all that apply.***

▢ Schedule swaps: resident requiring coverage arranges voluntary swaps

▢ Schedule swaps: program coordinates / requires swaps

*Display This Choice: If Q1 Does Not Equal No: we do not have a system in place*

▢ Use of back-up system

*Display This Choice: If Q1 = No: we do not have a system in place*

▢ Use of a *long-term* absence back-up system

*Display This Choice: If Q1 Does Not Equal No: we do not have a system in place*

▢ Use of *separate* long-term absence back-up system

▢ Schedule created around absences

▢ Other (please explain):

*Display This Question: If Q1 Does Not Equal No: we do not have a system in place*

**Q14 How often do your IM residents provide coverage when residents from other programs are unable to work their assignments on medicine services?**

- Always
- Most of the time
- Some of the time
- Rarely
- Never
- *Unsure*

*Display This Question: If Q1 Does Not Equal No: we do not have a system in place*

**Q15 How do you believe that use of your coverage system has changed *post-COVID-19 pandemic* (or since you were appointed as program director, if you have been in your position for less time)?**

- It has greatly increased
- It has somewhat increased
- It has remained the same
- It has somewhat decreased
- It has greatly decreased
- *Unsure / Do not know*

*Display This Question: If Q1 Does Not Equal No: we do not have a system in place*

**Q16 What has been the most challenging aspect(s) of back-up coverage for your program?**

*Display This Question: If Q1 = No: we do not have a system in place*

**Q17 What has been the most challenging aspect(s) of providing coverage when a resident is unable to work due to an unplanned / unexpected reason in your program?**

***You have completed Section II. Please proceed!***

**End of Block: Section II. Residency “Back-up, Sick Pull, Jeopardy” Coverage Systems**

**END OF DOCUMENT**

**Appendix 2**

Statistical Analysis: Strategies used to assess the statistical representativeness of the survey responses

To assess the statistical representativeness of the survey responses, we first identified variables (from the sources cited above) that demonstrated the most explanatory power with respect to residency program study eligibility (N=586). We used quantile (median) regression with “total number of filled ACGME-approved resident positions” as the dependent variable (pseudo R^2^=0.39; raw sum of deviations: 7,990.5), which is an essential characteristic for study eligibility (median regression was used because of the nonparametric distribution of filled positions). Program original accreditation year, accreditation status, type, U.S. Census region, total number of full-time physician faculty, offering preliminary resident positions, and offering J-1 visa sponsorship for residents demonstrated significance as predictor variables (p≤0.05).

We then conducted a second median regression model with “total filled ACGME-approved resident positions” for *survey-eligible* programs (n=466); the same variables as above also demonstrated significance as predictors for survey eligibility (pseudo R^2^=0.41; raw sum of deviations: 6,833.5). This confirmed that the survey-eligible population (n=466) was generally similar to the larger study-eligible population (N=586).

We compared survey respondents to the complete survey-eligible population using characteristics that explained most of the study population variance (i.e., independent variables from the regression models above, such as program original accreditation and accreditation status). We tested for possible associations between categorical variables using the Adjusted Wald (Pearson) Chi-Square test of association (with one degree of freedom).
